# [Corrigendum] Acquired resistance of pancreatic cancer cells to treatment with gemcitabine and HER-inhibitors is accompanied by increased sensitivity to STAT3 inhibition

**DOI:** 10.3892/ijo.2026.5948

**Published:** 2026-10-09

**Authors:** Nikolaos Ioannou, Alan M. Seddon, Angus Dalgleish, David Mackintosh, Flavio Solca, Helmout Modjtahedi

Int J Oncol 48: 908-918, 2016; DOI: 10.3892/ijo.2016.3320

Following the publication of the above article, an interested reader drew the authors' attention to the fact that, for the western blots shown in Fig. 5 on p. 914, the β-actin blots shown in the rightmost lanes for the 'BcPc3 OSIR' blots (the 'Crixotinib' and 'Af+Cr' lanes of the gel) were strikingly similar to the β-actin blots shown in Fig. 2B (for the 'BxPc3 parental' blots), albeit with horizontal and vertical resizing of the bands concerned.

Upon contacting the authors, they realized that Fig. 5 had inadvertently been assembled incorrectly (the same β-actin cropped image was used for both samples in question after the files had erroneously being saved under the same name). The authors were able to consult their original data, and provided the Editorial Office with access to the images of the relevant full-length original blots. The revised version of Fig. 5, now showing the correct data for the β-actin blots for the 'BcPc3 OSIR' experiments, is shown on the next page. Note that this error did not affect the overall conclusions reported in the study. The authors are grateful to the Editor of *International Journal of Oncology* for allowing them this opportunity to publish a Corrigendum, and all the authors agree with its publication. Furthermore, the authors thank the interested reader for drawing this error in their paper to their attention, and apologize to the readership for any inconvenience caused.

## Figures and Tables

**Figure 5.**
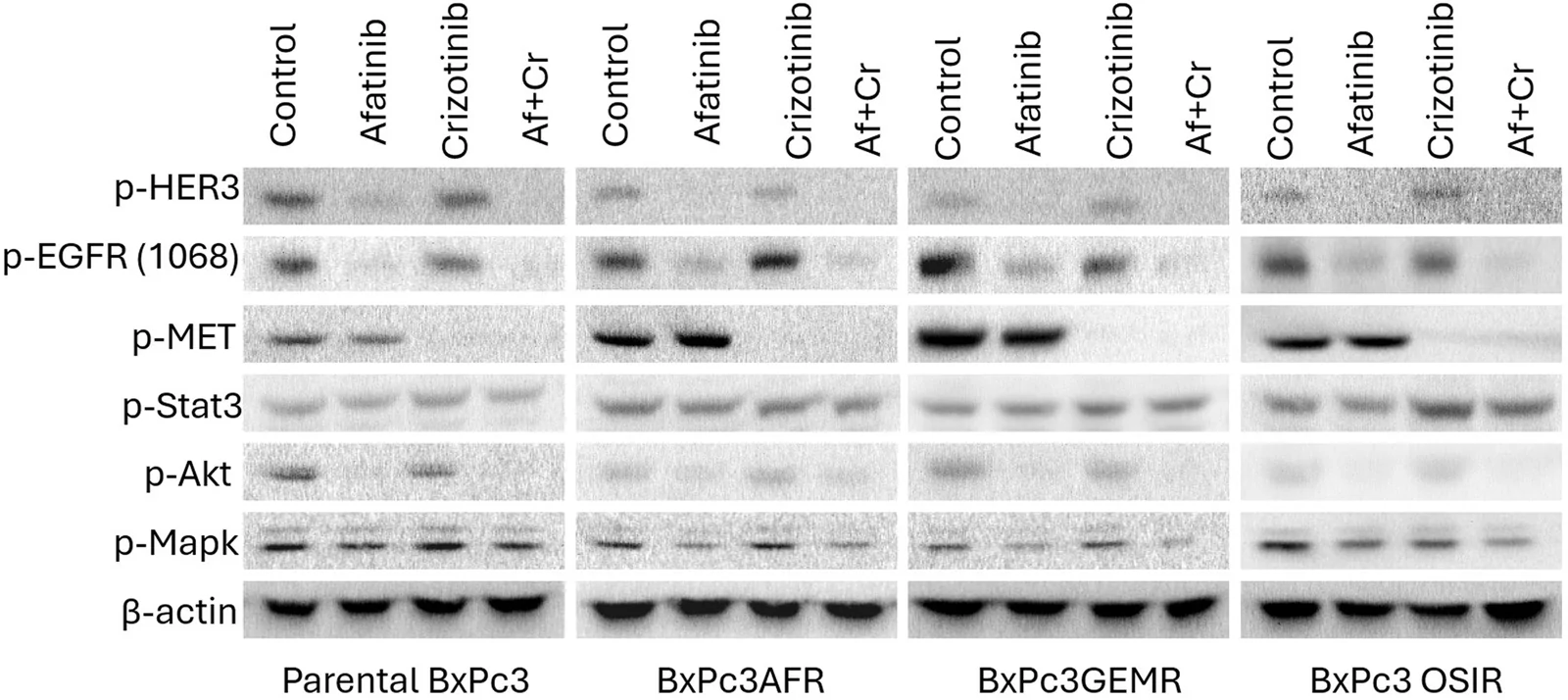
Basal levels of phosphorylated HER3, EGFR (Y1068) c-MET and downstream molecules AKT, MAPK and STAT3 in parental BxPc3 cells and drug-resistant cell lines BxPc3AFR, BxPc3GEMR and BxPc3OSIR either grown in 10% FBS supplemented growth medium alone or with Afatinib, Crizotinib or their combination. Cells were grown to near confluence in 10% FBS at 37°C, and incubated overnight. Cells were then lysed, proteins were separated by SDS-PAGE, transferred to PVDF membranes and probed with the antibodies of interest. Representative of two independent experiments.

